# Positive and Negative Impacts of Non-Native Bee Species around the World

**DOI:** 10.3390/insects7040069

**Published:** 2016-11-28

**Authors:** Laura Russo

**Affiliations:** Mueller Lab, Biology Department, Pennsylvania State University, University Park, PA 16802, USA; lar322@psu.edu

**Keywords:** bees, competition, genetic introgression, impacts, invasive species, pollination, species introductions

## Abstract

Though they are relatively understudied, non-native bees are ubiquitous and have enormous potential economic and environmental impacts. These impacts may be positive or negative, and are often unquantified. In this manuscript, I review literature on the known distribution and environmental and economic impacts of 80 species of introduced bees. The potential negative impacts of non-native bees include competition with native bees for nesting sites or floral resources, pollination of invasive weeds, co-invasion with pathogens and parasites, genetic introgression, damage to buildings, affecting the pollination of native plant species, and changing the structure of native pollination networks. The potential positive impacts of non-native bees include agricultural pollination, availability for scientific research, rescue of native species, and resilience to human-mediated disturbance and climate change. Most non-native bee species are accidentally introduced and nest in stems, twigs, and cavities in wood. In terms of number of species, the best represented families are Megachilidae and Apidae, and the best represented genus is *Megachile*. The best studied genera are *Apis* and *Bombus*, and most of the species in these genera were deliberately introduced for agricultural pollination. Thus, we know little about the majority of non-native bees, accidentally introduced or spreading beyond their native ranges.

## 1. Introduction

The accidental introduction of some invasive insects can decimate ecosystems [1] or cause billions of dollars of environmental [2], crop [3], or building damage [4]. The majority of these insects are not noted for their potential positive impacts. On the other hand, some insects have been deliberately introduced for the services they provide to humans, without full consideration for their potential negative impacts. These insects may spread beyond the areas where they have been deliberately introduced and even become invasive pests in some cases [5]. Bees are one such group of insects, often introduced for their pollination services, but also with the potential to have negative economic and environmental impacts.

Bee species have been both introduced accidentally and deliberately around the world. Perhaps the most well-known of these introduced bee species is the European Honeybee (*Apis mellifera*), the most managed bee in the world, but over 70 other species have become established outside of their native ranges. There is increasing concern about the potential negative effects of these introduced bees, and there have even been efforts to eradicate them in some areas [6]. However, most attention is focused on the highly abundant and widely introduced species, including *A. mellifera* and *Bombus terrestris* as well as the managed solitary bee, *Megachile rotundata*.

Most non-native bee species are accidentally introduced and the potential negative and positive impacts to their introductions have not been explored. Learning about the potential impacts of introduced bees is particularly important as new species are still being proposed for domestication, and ranges will continue to change naturally and through human transportation. The purpose of this review is to collate information on all recorded non-native bee species around the world, and to discuss some of the potential outcomes of their introductions. I reviewed over 450 papers (266 of those papers are cited in this manuscript: 145 in the main text and tables and the remainder in the supplementary tables) addressing the distributions and impacts of introduced bees around the world, and present recorded positive and negative impacts here.

## 2. Non-Native Bee Species

For the purposes of this paper, a non-native species is defined as having one or more populations outside of its historical range. Non-native species can have both negative and positive impacts [7]. Non-native species with recognized negative impacts which are difficult to control are often referred to as “invasive” and some bee species with non-native populations are referred to in this way. However, because of the widely recognized beneficial impacts of bees, and the fact that they are often deliberately introduced, not all non-native bee species are considered invasive. Thus, I will simply refer to them as non-native species here.

The literature reviewed by the author included 80 recorded non-native bee species from 30 genera around the world (Table 1). These non-native bees are unevenly distributed among the 7 bee families, suggesting attributes of some bee families might make them more likely to have adventive or invasive species. Melittidae and Stenotritidae are not represented by any recorded non-native species. The mining bee family, Andrenidae, is only represented by one non-native species (*Andrena wilkella*), while both Colletidae and Halictidae have 8 non-native representatives. Indeed, the non-native bees are dominated by representatives of the families Megachilidae (33 non-native species) and Apidae (30 non-native species), though some of the other families have fewer species overall and are thus less likely to have invasive representatives. With 13 non-native species, *Megachile* is the best represented genus.

The majority (73%) of the bee species with non-native populations were likely accidentally introduced, while a minority (18%) were deliberately introduced, and a small number (5%) naturally expanded their ranges. The introduction history of the remainder is still uncertain. The majority (69%) of all non-native bee species nest in stems, twigs, existing cavities, or holes they bore into wood, while a smaller number (26%) nest in the ground, and very few (5%) have exposed nests. The proportion of bees that nest in stems, twigs, or other cavities increases to 77% when only accidentally introduced species are considered. Ten of the non-native species are suspected to be oligolectic and have likely been transported with their plant hosts. There are only two cleptoparasitic species represented among the non-native bees, though some non-native *Sphecodes* have been found in Hawaii (*pers. comm*. S. Droege).

Islands have the largest number of introduced bees, and indeed, 27 of the 80 non-native bee species are only non-native on islands (not counting Australia as an island). These non-native bee species sometimes become the most diverse and abundant component of the otherwise depauperate bee fauna of island ecosystems. For example, the Galapagos Islands have only one native bee species, but two non-native bee species have been introduced [22]. In the southwest Pacific islands of Fiji, Samoa, and Vanuatu, there is some evidence to indicate that most, if not all, apid and megachilid bees have been introduced by humans [26,64]. A similar situation likely exists in French Polynesia [39]. On the Hawaiian Islands, which likely have only 69 native bee species, there are 14 non-native bee species recorded [9,17].

There are likely many more unrecorded introduced bee species. This list is dominated by species introduced to North America; 34 of the 80 species are only reported as non-native in North America, including Hawaii (25 excluding Hawaii). A total of 55 bee species (69%) have been recorded as introduced to North America and it is unclear whether North America is truly more vulnerable to invasion or whether there is a sampling bias. As a contrast, there are relatively few non-native bees recorded in the well-studied European bee fauna.

## 3. Impacts

**Figure 1:**
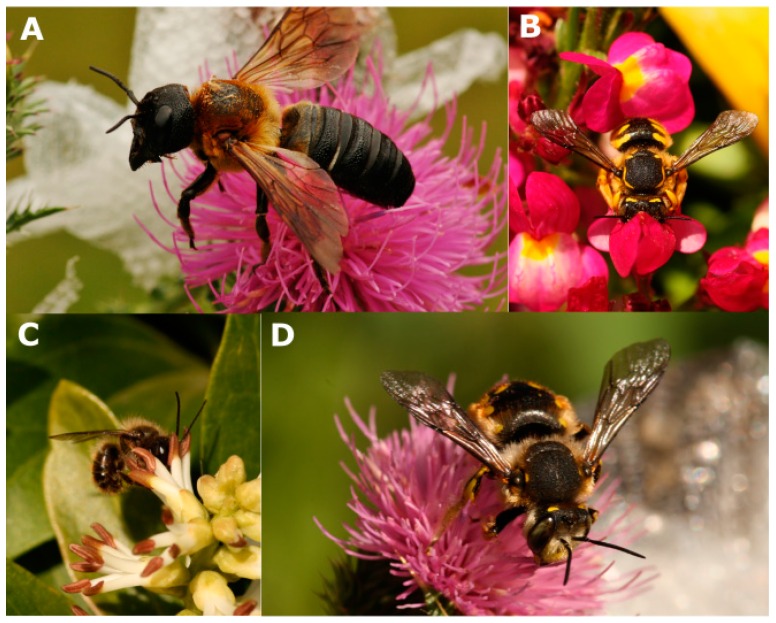
Non-native bees on non-native plants, including (**A**) *Megachile sculpturalis* on *Carduus acanthoides*; **(B**) *Anthidium manicatum* on *Linaria reticulata*; (**C**) *Osmia cornifrons* on *Pachysandra terminalis*; and (**D**) *Anthidium manicatum* on *Carduus acanthoides*. Photographs by the author.

Outside of the genera *Apis* and *Bombus*, empirical evidence for impacts is sparse; for 25 species researchers have suggested hypothetical impacts, but have not measured impacts empirically. For an additional 29 species, there are neither hypothetical nor empirical impacts in the literature. For 13 species, there are potential negative impacts, but no potential positive impacts recorded in the literature, and, conversely, there are 11 species for which only potential positives are recorded. Thus, for more than two thirds of the non-native bee species distributed around the world, we have no empirical evidence of any impact of their introduction. This is not equivalent to evidence for the absence of an impact, though such evidence would be less likely to be published [65]. Moreover, it is more difficult to obtain evidence for some impacts than others. For example, it is easier to demonstrate that a non-native bee can pollinate an invasive weed than to show it competes with native bees, as the former can be directly measurable (Figure 1).

## 4. Negative Impacts

Goulson [19] conducted a review of the potential negative impacts of 17 non-native bees, which he listed as competition for floral resources or nesting sites, transmission of pathogens or parasites, affecting the seed set of native plants, and pollinating invasive plant species. However, many bee species were introduced after 2000 (Figure S1) and several additional negative impacts have been mentioned in the literature, including alteration of pollination networks, damage to buildings, and genetic introgression through hybridization of managed populations/species with wild populations/species.

### 4.1. Apis/Bombus

Because entire reviews have been written just on the impacts of the introduced species from the *Apis* and *Bombus* genera (e.g., [66,67,68,69]), I have collated selected references for these genera in separate tables in the supplementary materials. Despite the abundance of research on these two genera, the literature is still mixed on their potential impacts and the results of many studies are either inconclusive or contradictory [66]. For example, there is both abundant evidence that honeybees can reduce the pollination of native plants, and also that they can improve the pollination of native plants (Tables S1 and S2). Some also argue that introduced *Apis* only have negative impacts on other members of their own genus [70]. The negative impact of genetic introgression has only been recorded for *Apis/Bombus*, and there seems to be some strong empirical support for this potential outcome in *Bombus terrestris*. Though there is some empirical support for all negative impacts (except damage to buildings) across these two genera, there are also some studies that record the absence of competition, pollination of invasive weeds, and decreasing the fitness of native plants. The only category where there is plenty of empirical support and no contradicting studies is the spread of parasites and pathogens introduced along with *Apis/Bombus* species (Table S1).

### 4.2. Other Genera

The strongest evidence for negative impacts in non-*Apis/Bombus* species is in competition for floral resources, transmission of parasites and pathogens, and pollination of invasive weeds. However, there is little empirical evidence that these non-native bees compete for floral resources and there is empirical evidence demonstrating a lack competition in several cases (Table 2). There only seems to be evidence for nesting competition with native species in the genus *Megachile* and the potential economic negative of damage to buildings has only been hypothetically recorded for *Lithurgus chrysurus* [71,72], though some other wood boring species may also have this potential. There is no empirical support for the potential of these non-*Apis/Bombus* species to degrade pollination networks or to negatively affect the pollination of native plant species, but there is some concern in the literature that several species will have this effect, particularly on islands [26].

If hypothetical impacts can be considered a measure of the relative amount of concern about the introduction of a given species, the European Wool Carder bee (*Anthidium manicatum*), is the most concerning of the non-*Apis/Bombus* introduced bees. Indeed, the range of this species has increased rapidly in recent years, and it seems to be approaching a global distribution [73]. There is evidence that the non-native *Osmia cornifrons* and *O. cornuta* and their native congener *O. lignaria* can share some parasites [74,75], though the potential for the introduced bee to spread the parasite to native bee species has not been explored. In contrast, the most empirical evidence for negative impacts seems to be for the globally introduced managed Alfalfa Leaf Cutter Bee (*Megachile rotundata*) and the accidentally introduced Taiwanese bamboo carpenter bee (*Xylocopa tranquebarorum*).

## 5. Positive Impacts

To the best of my knowledge the positive impacts of introduced bees have not been reviewed although the pollination services provided by diverse communities of bees are well known [98,99,100,101] and the interactions between native and non-native bees might even be beneficial in some cases [102]. Non-native bees can have positive impacts by pollinating agricultural crops, acting as biological control agents, rescuing native plant species whose native pollinators have been lost, increasing resilience to human disturbance and climate change, encouraging scientific investigations through lab rearing, serving as bio-indicators, and promoting the study of natural history.

### 5.1. Apis/Bombus

As with negative impacts, the positive impacts of the *Apis* and *Bombus* genera are reviewed in the supplemental materials (Table S2). They can have the same positive impacts as the other bee genera and, as with the negative impacts, are better studied in general. The majority of these bees were deliberately introduced for their pollination services to agriculture, which are widely recognized. However, their ease of management has also led to their utility as study species for scientific research. They have also been demonstrated to rescue native plant species when their native pollinators are in decline, though their role in the decline of these native pollinators is unclear (Table S1). It is also possible that their impacts on native pollinators are confounded with human disturbance, as several of these species are more resilient to human impacts.

### 5.2. Other Genera

In the non-*Apis/Bombus* genera, the strongest evidence for positive impacts comes unsurprisingly from the potential to be agricultural pollinators. There is likely a publication bias for this effect, as it confers an economic benefit to humans [103]. Several non-native bee species were deliberately introduced for their pollination services (Table 1), but this is also listed as a hypothetical benefit for many accidentally introduced bee species (Table 3).

Because deliberately introduced species are generally easy to manage and rear in a laboratory setting, many of these provide an additional benefit to science and are often used for pesticide, genetic, and behavioral studies. In the case of *Osmia cornuta*, they have even shown to be potentially useful for the application of biocontrol to manage invasive pest species in crops [13] or bioindicators for environmental quality [104]. Many other bee species have the potential to provide this benefit, particularly those that readily nest in hollow tubes as a high proportion of introduced bees do.

Finally, many of these non-native species have been accidentally spread by humans because they flourish in human-modified landscapes. These bee species have the potential to supplement pollination services where native species have been lost and demonstrate resilience to human disturbance and climate change. Indeed, several of the listed species have only been recorded in urban areas (e.g., *Anthidium oblongatum* [105], *Pseudanthidium nana* (*pers. comm.* S. Droege)). Bees capable of handling human disturbance and habitat degradation might not only provision crop species with pollination services, but also have the potential to rescue native plant species whose more sensitive native pollinators have been lost [106], though this has not yet been listed as a potential benefit of non-*Apis/Bombus* genera.

## 6. Discussion

Though much research has been done on the impacts of bees in the *Apis* and *Bombus* genera, much less is known about the impacts of non-native bees in other genera. This is partly due to the fact that many of these bees have either only recently been introduced (e.g., [24]), or only recently been discovered as non-native [11]. It is particularly important to understand these impacts as bees continue to be accidentally introduced by humans, and expand their distributions in response to disturbance and climate change. As other authors have noted (e.g., [19]), conducting experiments to demonstrate some of the aforementioned impacts can be challenging, but with multiple studies in disparate locations, we can get a better understanding of the overall context dependence of negative and positive species impacts.

Future research should explore the impacts of bees beyond their roles as pollinators or competitors in ecological communities, for example, as prey in altered trophic webs. A sudden influx of a highly abundant food source, even if it is novel, might have implications for some predators [137]. It may change the population dynamics of insectivorous birds, generalist insect predators, or insects that prey solely on bees, such as beewolves [138]. There may also be interesting impacts of non-native bees on public perception of important ecological issues [139].

As is sometimes the case with introduced species [140], some of the bee species that are considered invasive in their introduced range have become quite rare in their native range, such as *Bombus ruderatus* [141] and *B. subterraneous* [142]. The management options for these bees in their introduced range are more complicated because conservation in their native range is also a concern. Indeed, some breeding programs for reintroduction of these *Bombus* species are being developed in their non-native range in New Zealand [143].

## 7. Conclusions

Invasive non-native bees present a complex topic because their negative impacts may be inextricably tied to the pollination services they provide to humans and the potential positive impacts they have in their role as pollinators. Whether or not they can have a dramatic effect on native communities and ecosystems or whether anthropogenic impacts such as habitat degradation are driving changes in community structure is unknown and probably understudied [85,144]. However, concerns about non-native bees exist, especially when their ranges expand rapidly, and for the majority of introduced bee species little or nothing is known. Thus, their role in novel ecosystems should be addressed, weighing both positive and negative influences they have on native species.

## Figures and Tables

**Table 1:** A list of non-native bee species around the world, sorted by family and including their probable method of introduction (deliberately introduced: I; accidentally introduced: A; naturally expanding or shifting range: N), year of introduction, origin, and known areas of introduction. Question marks (?) represent uncertainty in the timing or distribution of introduced and native range. This often occurs when the bee fauna of a given area has only recently been studied and in some cases the non-native species has potentially been present for a long time.

Colletidae (8)	Non-native Species		Year	From	Found in	Reference
	*Chilicola rostrata*	A	2008	Argentina	Chile	[8]
	*Hylaeus (Prosopis) variegates*	A	1990	North Africa	New York City	*pers. comm.* S. Droege
	*Hylaeus albonitens*	A	1995	Australia	Hawaii	[9]
	*Hylaeus hyalinatus*	A	1990	Europe	New York City, S Ontario, New Jersey, Pennsylvania	[10,11]
	*Hylaeus leptocephalus*	A	1900	Europe	US, S Canada	[9,11,12]
	*Hylaeus punctatus*	A	1980	Europe	US, Chile, Canada, Argentina, Brazil	[11]
	*Hylaeus strenuus*	A	2007	Asia	Hawaii	[13,14]
	*Hyleoides concinna*	A	1980	Australia	New Zealand	[15]
Andrenidae (1)						
	*Andrena wilkella*	A	1900s	Europe and N Asia	NE US and S Canada	[11,12,16]
Halictidae (8)						
	*Halictus tectus*	A	2000	Europe to Mongolia	Philadelphia, Baltimore, Washington, DC	[12]
	*Lasioglossum eleutherense*	A	1990	Bahamas and Cuba	Florida	*pers. comm.* S. Droege
	*Lasioglossum imbrex*	A	2013	US	Hawaii	[17,18]
	*Lasioglossum impavidum*	A	2003	W US	Hawaii	[9]
	*Lasioglossum leucozonium*	A	1900s	Europe and North Asia	US and S Canada	[11,16]
	*Lasioglossum microlepoides*	A	2013	continental US	Hawaii	[17,18]
	*Lasioglossum zonulum*	A	?	Europe and S China	North America	[11]
	*Nomia melanderi*	I	1970	North America	New Zealand	[19]
Megachilidae (33)						
	*Afranthidium (Immanthidium) repetitum*	A	2000	Africa	Australia	[20]
	*Anthidium manicatum*	A	1960	Europe, N Africa, Near East	Chile, Brazil, Argentina, Uruguay, the US, Canada, New Zealand, Siberia, Peru, Suriname, Paraguay	[11,12,16]
	*Anthidium oblongatum*	A	1990	Europe and Near East	NE US and S Canada	[11,12]
	*Anthidium vigintiduopunctatum*	A	2006	South America, Ecuador, Peru	Galapagos, Fiji?	[21,22]
	*Chelostoma campanularum*	A	1960	Europe and Near East	New York, Connecticut, and S Ontario	[11,23]
	*Chelostoma rapunculi*	A	1960	Europe and Near East	New York and S Ontario	[11,23]
	*Coelioxys coturnix*	A	2000	Europe, North Africa, Mediterranean, India?	E US	*pers. comm.* S. Droege
	*Heriades truncorum*	A	2010	Europe and Near East	Maryland	*pers. comm.* S. Droege
	*Hoplitis adunca*		2016	Europe, Asia, Africa	Britain	[24]
	*Hoplitis anthocopoides*	A	1960	Europe	US, S Ontario	[11,25]
	*Lithurgus bractipes*	A	?	?	Fiji	[26]
	*Lithurgus chrysurus*	A	1970	Europe, Near East, N Africa	Pennsylvania and New Jersey	[12]
	*Lithurgus huberi*	A	1907	Asia	South America, Argentina	[27,28,29]
	*Lithurgus scabrosus*	A	1907	Europe	Hawaii, Vanuatu	[9]
	*Megachile apicalis*	A	1930	Europe, N Africa, Near/Middle East	US, Canada	[11,12]
	*Megachile australis*	A		SE Asia	Vanuatu, Samoa	[26]
	*Megachile chlorura*	A	1988	Philippines	Hawaii	[9]
	*Megachile concinna*	A	1940	Africa	West Indies, Mexico, US, Argentina	[12]
	*Megachile ericetorum*	A	2000	Europe, Near East, China	S Ontario and New York	[11]
	*Megachile fullawayi*	A	1921	Guam	Hawaii	[30]
	*Megachile gentilis*	A	?	W US	Hawaii	[9,31]
	*Megachile lanata*	A	1700–1800	India and China	West Indies and N South America, Florida, Antilles, Hawaii	[32]
	*Megachile rotundata*	I,A	1920–1940	Europe to China	North America to N Mexico, New Zealand, Chile, Argentina, Australia, Canada, Denmark	[11,12,19,33]
	*Megachile rufipennis*	A	1511–1867	Old World	Antilles	[32]
	*Megachile sculpturalis*	A	1990	Far east China, Korea, Japan	US, S Canada, Europe	[11,12,34,35]
	*Megachile timberlakei*	A	2010	Hawaii?	Galapagos	[9,22,36]
	*Megachile umbripenne*	A	2013	S Asia	Fiji, Samoa, Hawaii?	[9,26]
	*Osmia caerulescens*	A	1800s	Europe, N Africa, Near East, India	US, S Canada, New Zealand	[11,12]
	*Osmia cornifrons*	I	1960	East China, Japan	US, Denmark, Korea	[12]
	*Osmia cornuta*	I	1980	Europe, N Africa, Near East	establishment not documented	[37]
	*Osmia ribifloris*	I	1991	W US	Maine/E US, establishment uncertain	[38]
	*Osmia taurus*	A	2000	East China, Japan	E US, Michigan	[16] Gibbs et al *in prep*
	*Pseudoanthidium nana*	A	2000	Europe and Near East	NE US	*pers. comm.* S. Droege
Apidae (30)						
	*Amegilla pulchra*	A	?	Australia	Fiji	[39]
	*Anthophora villosula*	I	1980	Japan	E US	[12]
	*Apis cerana*	A	2007	Asia	Australia, Russia, Iran (Crane 1995), Papua New Guinea (Bradbear and MacKay 1995), Samoa, Fiji, Vanuatu	[40]
	*Apis dorsata*	A	?	Asia	Japan	[41]
	*Apis florea*	I	1985	Oman, Asia, Indonesia	Iraq, Sudan (Glaiim 2005)	[42,43,44,45]
	*Apis mellifera*	I	1620	N Europe, Meditteranean	globally introduced	[11,12]
	*Bombus hortorum*	I	1885	UK	New Zealand	[19]
	*Bombus hypnorum*	N	2001	Europe	UK	[46,47]
	*Bombus impatiens*	I	2003	North America	Chile, Mexico, Central America, Canada	[33,48]
	*Bombus lucorum*		1981	Europe, China	Iceland	[19]
	*Bombus ruderatus*	I	1885	UK	New Zealand, Chile, Argentina, Patagonia, Canary Islands	[19,33,49]
	*Bombus subterraneous*	I	1885	UK	New Zealand	[19]
	*Bombus terrestris*	I	1885	UK	Chile, China, Israel, Japan, Mexico, South Africa, South Korea, New Zealand, Tasmania, and Taiwan	[19,33,49,50,51]
	*Braunsapis puangensis*	A	2003	Asia, india	Fiji	[52,53,54,55]
	*Centris nitida*	A	2000	SW US, Mexico, central, S America	Florida	[56]
	*Ceratina arizonensis*	A	1950	W US	Hawaii	[9]
	*Ceratina cobaltina*	A	1970	Mexico	Texas	*pers. comm.* S. Droege
	*Ceratina dallatorreana*	A	1940	Meditteranean	California	[57]
	*Ceratina dentipes*	A	1909	Turkey, Cyprus, S Asia, Australia	Vanuatu, Fiji, Samoa, Cook Islands, Hawaii, Japan, Mauritius	[9]
	*Ceratina smaragdula*	I	1960	Pakistan, India, SE Asia	Hawaii, Australia	[9]
	*Euglossa dilemma*	A	2000	Mexico and Central America	Florida	[58]
	*Peponapis pruinosa*	N	?	Mexico	North America	[59]
	*Plebia frontalis*	I?	2010	Mexico, Central, South America	California	*pers. comm.* S. Droege
	*Triepeolus remigatus*	N	?	Mexico	North America	*pers. comm.* S. Droege
	*Xenoglossa strenua*	N	?	Mexico	North America	*pers. comm.* S. Droege
	*Xylocopa appendiculata*	A	2010	Japan and China	California	[60]
	*Xylocopa augusti*	A	2013	Argentina	Chile	[61]
	*Xylocopa sonorina*	I	?	W US	Samoa, Hawaii, Guam, Northern Marianas Islands, Japan	[9,41,62]
	*Xylocopa tabaniformis parkinsoniae*	A	1990	South Texas	W US	*pers. comm.* S. Droege
	*Xylocopa tranquebarorum*	A	2005	Asia	Japan	[63]

**Table 2:** Potential negative impacts of non-*Apis* or *Bombus* species, including competition for nesting sites and floral resources, co-introduction with pathogens or parasites, pollination of invasive weeds, alteration of resident pollination networks, damage to buildings, and changing pollination of native plant species. Bold and underlined text refers to citations with an empirical component while unbolded text refers to papers that refer to impacts only from a hypothetical standpoint. Light grey shading indicates species for which neither positive nor negative impacts have been recorded, while dark grey indicates species for which only positive impacts have been recorded. “But see” refers to manuscripts that show evidence or describe the opposite of the effect.

Non-native Species	Nesting Sites	Floral Resources	Pathogens/Parasites	Invasive Weeds	Alteration of Pollination Networks	Damage to Buildings	Change Pollination
**Colletidae**							
*Chilicola rostrata*							
*Hylaeus albonitens*		[76]					
*Hylaeus hyalinatus*				[77]			
*Hylaeus leptocephalus*							
*Hylaeus punctatus*							
*Hylaeus strenuus*		[14]					
*Hylaeus (Prosopis) variegates * ^¥^							
*Hyleoides concinna*	[15]						
**Andrenidae**							
*Andrena wilkella*							
**Halictidae**							
*Halictus tectus*							
*Lasioglossum eleutherense*							
*Lasioglossum imbrex*							
*Lasioglossum impavidum*		[76]					
*Lasioglossum leucozonium*							
*Lasioglossum microlepoides*							
*Nomia melanderi*							
**Megachilidae**							
*Afranthidium repetitum*							
*Anthidium manicatum*		[73] but see [78]	[73]	[73,78]			
*Anthidium oblongatum*							
*Anthidium vigintiduopunctatum*							
Chelostoma campanularum **							
Chelostoma rapunculi **							
*Coelioxys coturnix*							
*Heriades truncorum*							
*Hoplitis adunca*							
Hoplitis anthocopoides **							
*Lithurgus bractipes*					[26]		
Lithurgus chrysurus **						[71,72]	
Lithurgus huberi **							
*Lithurgus scabrosus*					[26]		
Megachile apicalis **	[19,79],[**80**]			[**81**]			
*Megachile australis*					[26]		
Megachile concinna **			[**82**]				
*Megachile ericetorum*							
*Megachile fullawayi*							
*Megachile gentilis*							
*Megachile lanata*							
Megachile rotundata **	[79] but see [83],[**84**],[85]	But see [**84**]	[19],[**86**]	[19] but see [**84**]			
*Megachile rufipennis*							
*Megachile sculpturalis*	[87,88]			[89,90]	[90]		
*Megachile timberlakei*	[22]	[22]			[22]		[22]
*Megachile umbripenne*					[26]		
*Osmia caerulescens*							
*Osmia cornifrons*			[**86**]				
*Osmia cornuta* ^¥^							
*Osmia ribifloris*							
*Osmia taurus*							
Pseudoanthidium nana **							
**Apidae**							
*Amegilla pulchra*				[39]			
*Anthophora plumipes*							
*Braunsapis puangensis*		[52]		[28,52],[**91**]			
*Centris nitida*		But see [**56**]		[**92**]			
*Ceratina arizonensis*		[76]					
*Ceratina cobaltina* ^¥^							
*Ceratina dallatorreana*							
*Ceratina dentipes*		[76]					
*Ceratina smaragdula*		[76]					
*Euglossa dilemma*				[**93,94**]			
Peponapis pruinosa **							
*Plebia frontalis* ^¥^							
**Triepeolus remigatus**							
*Xenoglossa strenua*							
*Xylocopa appendiculata*			[60]				
*Xylocopa augusti*							
*Xylocopa sonorina*				[39]			[62,95]
*Xylocopa tabaniformis parkinsoniae*							
*Xylocopa tranquebarorum*			[**63,96,97**]				

** oligolectic; ^¥^ establishment uncertain.

**Table 3:** Potential positive impacts of non-*Apis* or *Bombus* species, including agricultural pollination, biocontrol of pest species, ability to encourage scientific research in lab-reared studies, as bioindicators, or for studies of natural history, and resilience to human disturbance and climate change. Bold and underlined text refers to citations with an empirical component while unbolded text refers to papers that refer to impacts only from a hypothetical standpoint. Light grey shading indicates species for which neither positive nor negative impacts have been recorded, while dark grey indicates species for which only negative impacts have been recorded. “But see” refers to manuscripts that show evidence or describe the opposite of the effect.

Non-native Species	Agricultural Pollination	Biocontrol	Lab Reared	Bioindicators	Natural History	Resilience
**Colletidae**						
*Chilicola rostrata*						
*Hylaeus albonitens*						
*Hylaeus hyalinatus*						[77]
*Hylaeus leptocephalus*						
*Hylaeus punctatus*						
*Hylaeus strenuus*						
*Hylaeus (Prosopis) variegates * ^¥^						
*Hyleoides concinna*						
**Andrenidae**						
*Andrena wilkella*	[31]					[107]
**Halictidae**						
*Halictus tectus*						
*Lasioglossum eleutherense*						
*Lasioglossum imbrex*						
*Lasioglossum impavidum*						
*Lasioglossum leucozonium*	[108] *					
*Lasioglossum microlepoides*						
*Nomia melanderi*	[**103**],[109,110]					
**Megachilidae**						
*Afranthidium repetitum*						
*Anthidium manicatum*			[111]			
*Anthidium oblongatum*						
*Anthidium vigintiduopunctatum*					[26]	
Chelostoma campanularum **						
Chelostoma rapunculi **						
*Coelioxys coturnix*						
*Heriades truncorum*			[112]			
*Hoplitis adunca*						
Hoplitis anthocopoides **						
*Lithurgus bractipes*	[26]					
Lithurgus chrysurus **						
Lithurgus huberi **						
*Lithurgus scabrosus*	[26]					
Megachile apicalis **						
*Megachile australis*	[26]					
Megachile concinna **	[**113**]					
*Megachile ericetorum*						
*Megachile fullawayi*						
*Megachile gentilis*	[31]					
*Megachile lanata*						
Megachile rotundata **	[26,31],[**38**],[73,109,114],[**115**],[116]		[**116,117,118,119**]		[**120**]	
*Megachile rufipennis*						
*Megachile sculpturalis*						
*Megachile timberlakei*						
*Megachile umbripenne*	[26]					
*Osmia caerulescens*						
*Osmia cornifrons*	[109,114,121],[**122,123,124**]		[**125,126,127**]			
*Osmia cornuta * ^¥^	[109],[**128**],[129]	[**130**]	[**131**]	[**104**]		
*Osmia ribifloris*	[19],[**38**]					
*Osmia taurus*						
Pseudoanthidium nana **						
**Apidae**						
*Amegilla pulchra*						
*Anthophora plumipes*	[132]					
*Braunsapis puangensis*	[52,133]				[52,53,133]	
*Centris nitida*						[56]
*Ceratina arizonensis*						
*Ceratina cobaltina * ^¥^						
*Ceratina dallatorreana*						
*Ceratina dentipes*						
*Ceratina smaragdula*	[134]					
*Euglossa dilemma*					[135]	
Peponapis pruinosa **	[59]					
*Plebia frontalis * ^¥^						
*Triepeolus remigatus*						
*Xenoglossa strenua*	[**136**]					
*Xylocopa appendiculata*						
*Xylocopa augusti*						
*Xylocopa sonorina*	[31]					
*Xylocopa tabaniformis parkinsoniae*						
*Xylocopa tranquebarorum*						

* Dissertation; ** oligolectic; ^¥^ establishment uncertain.

## Supplementary Materials

The following are available online at http://www.mdpi.com/2075-4450/7/4/69/s1. Table S1: Selected references of potential negative impacts of *Apis* or *Bombus* species; Table S2: Selected references for potential positive impacts of *Apis* or *Bombus* species. Figure S1: Frequency histogram of first collection records for introduced bee species. Note that dates of introduction are approximated and for most species have not been carefully studied.
