# Supplementary material for: Positive and Negative Impacts of Non-Native Bee Species around the World
Source: Insects. 2016 Nov 28;7(4):69. doi: 10.3390/insects7040069 (PMC5198217; doi:10.3390/insects7040069)
Supplement: Supplementary file 1 [file insects-07-00069-s001.pdf]

insects

MDPI

Supplementary Materials: Positive and Negative Impacts of Non-Native Bee Species around the World

Laura Russo

Table S1. Selected references of potential negative impacts of *Apis* or *Bombus* species. Bold, underlined, and shaded text refers to citations with an empirical component while unbolded text refers to papers that refer to impacts only from a hypothetical standpoint. Light grey shading indicates species for which neither positive nor negative impacts have been recorded. "But see" refers to manuscripts that show evidence or describe the opposite of the effect and is capitalized when only contradictory studies could be found. Note that *Apis mellifera scutellata* (the "Africanized" honeybee), is treated separately given the abundance of research specifically studying that subspecies.

Non-native Species	Nesting Sites	Floral Resources	Pathoens/ Parasites	Invasive Weeds	Altering Pollination Webs	Introgression	Decrease Plant Fitness
Apis cerana	[1]	[2]	[1–3]			[4]	
Apis dorsata							
Apis florea		[5]	[5]				
Apis mellifera	But see [6,7]	**[8–19]** but see [6,20–22]	**[9,23–26]**	**[27–35]** but see [6]	[36–38] **[39–43]** but see **[44]**	[4]	**[37,45]** [38,46,47] **[48,49]** but see **[50]**
Apis mellifera scutellata		**[51]** but see **[52–54]**			**[55–57]**		
Bombus hortorum				**[58,59]**			
Bombus hypnorum	But see **[60]**	But see **[60]**	**[61]**				
Bombus impatiens	[62]	[62,63]	[26,64–66]			[62]	
Bombus lucorum							
Bombus ruderatus		[67,**68**]	[69,70]	**[28,58,59,69,71,72]**	[36,39]		[39] but see **[73]**
Bombus subterraneous				**[59]**			
Bombus terrestris	[74–76]	**[67,70,74,75,77–84]** but see **[85,86]**	**[25,26,70,76,87–90]**	**[29,58,72,91–95]** but see [96]	**[38,39,68,81,97,98]**	**[4,76,88,99,100]**	**[47,76,49,86,97,101–103]**

Table S2. Selected references for potential positive impacts of *Apis* or *Bombus* species. Bold, underlined, and shaded text refers to citations with an empirical component while unbolded text refers to papers that refer to impacts only from a hypothetical standpoint. Light grey shading indicates species for which neither positive nor negative impacts have been recorded. "But see" refers to manuscripts that show evidence or describe the opposite of the effect. Note that *Apis mellifera scutellata* (the "Africanized" honeybee), is treated separately given the abundance of research specifically studying that subspecies.

Non-native Species	Agricultural Pollination	Lab Reared Studies	Natural History	Rescue of Native Species	Resilience to Human Disturbance and Climate Change
Apis cerana	[**1,104,105**]			[**106**]	
Apis dorsata					
Apis florea	[**5,107**]				
Apis mellifera	[7,108–112] but see [**15,113,114**]	[**115–119**]		[9] [**6,106,120–122**]	[**123,124**]
Apis mellifera scutellata	[125,126]				[**127**]
Bombus hortorum	[128,129] *	[**130,131**]	[**132,133**]		
Bombus hypnorum	[134]				[**60**,135]
Bombus impatiens	[**136**]	[**137**]			[138]
Bombus lucorum					
Bombus ruderatus	[139,**140,141**] *	[**130,131**]	[**132,133, 140,141**]	[**122**]	
Bombus subterraneous	[128,129] *		[**132,133**]		
Bombus terrestris	[68,76,94,142] but see [128,129] * [143]	[**131,144,145**]	[**132,133**]	[**122**]	[**146**]

* Dissertation

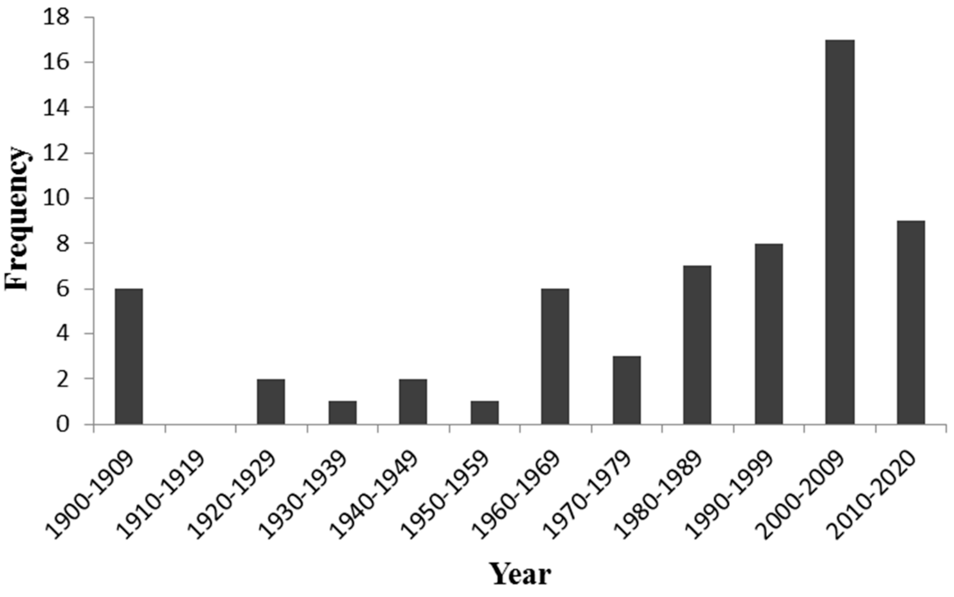

Figure S1. Frequency histogram of first collection records for introduced bee species. Note that dates of introduction are approximated and for most species have not been carefully studied.

References

1. Koetz, A. Ecology, behaviour and control of Apis cerana with a focus on relevance to the Australian incursion. *Insects* **2013**, *4*, 558–592.
2. Groom, S.; Schwarz, M. Bees in the Southwest Pacific: Origins, diversity and conservation. *Apidologie* **2011**, *42*, 759–770.
3. Oldroyd, B. Coevolution while you wait: Varroa jacobsoni, a new parasite of western honeybees. *Trends Ecol. Evol.* **1999**, *14*, 312–315.
4. Byatt, M.; Chapman, N.; Latty, T.; Oldroyd, B. The genetic consequences of the anthropogenic movement of social bees. *Insectes Soc.* **2016**, *63*, 15–24.
5. Moritz, R. F. A.; Haddad, N.; Bataieneh, A.; Shalmon, B.; Hefetz, A. Invasion of the dwarf honeybee Apis florea into the near East. *Biol. Invasions* **2010**, *12*, 1093–1099.
6. Huryn, V. Ecological impacts of introduced honey bees. *Q. Rev. Biol.* **1997**, *72*, 275–297.
7. Howlett, B.; Donovan, B. A review of New Zealand's deliberately introduced bee fauna: Current status and potential impacts. *N. Z. Entomol.* **2010**, *33*, 92–101.
8. Sugden, E. A.; Pyke, G. H. Effects of honey bees on colonies of Exoneura asimillima, an Australian native bee. *Aust. J. Ecol.* **1991**, *16*, 171–181.
9. Goulson, D. Effects of introduced bees on native ecosystems. *Annu. Rev. Ecol. Evol. Syst.* **2003**, *34*, 1–26.
10. Paini, D.; Roberts, J. Commercial honey bees (Apis mellifera) reduce the fecundity of an Australian native bee (Hylaeus alcyoneus). *Biol. Conserv.* **2005**, *123*, 103–112.
11. Yang, G. H. Harm of introducing the western honeybee Apis mellifera L. to the Chinese honeybee Apis cerana F. and its ecological impact. *Acta Entomol. Sin.* **2005**, *3*, 15.
12. Semida, F.; Elbanna, S. Impact of introduced honey bees on native bees at St. Katherine protectorate, South Sinai, Egypt. *Int. J. Agric.* **2006**, *8*, 191–194.
13. Magnacca, K. N. Conservation status of the endemic bees of Hawai'i, Hylaeus (Nesoprosopis) (Hymenoptera: Colletidae). *Pac. Sci.* **2007**, *61*, 173–190.
14. Goulson, D.; Sparrow, K. Evidence for competition between honeybees and bumblebees; effects on bumblebee worker size. *J. Insect Conserv.* **2009**, *16*, 177–181.
15. Badano, E.; Vergara, C. Potential negative effects of exotic honey bees on the diversity of native pollinators and yield of highland coffee plantations. *Agric. For. Entomol.* **2011**, *13*, 365–372.
16. Hudewenz, A.; Klein, A. Competition between honey bees and wild bees and the role of nesting resources in a nature reserve. *J. Insect Conserv.* **2013**, *17*, 1275–1283.
17. Cane, J.; Tepedino, V. Gauging the effect of honey bee pollen collection on native bee communities. *Conserv. Lett.* **2016**, doi:10.1111/conl.12263.
18. Herbertsson, L.; Lindström, S.; Rundlöf, M. Competition between managed honeybees and wild bumblebees depends on landscape context. *Basic Appl. Ecol.* **2016**, *17*, 609–616.
19. Torné-Noguera, A.; Rodrigo, A.; Osorio, S. Collateral effects of beekeeping: Impacts on pollen-nectar resources and wild bee communities. *Basic Appl. Ecol.* **2016**, *17*, 199–209.
20. Donovan, B. Interactions between native and introduced bees in New Zealand. *N. Z. J. Ecol.* **1980**, *3*, 104–116.
21. Roubik, D.W. Experimental community studies: Time-series tests of competition between African and Neotropical bees. *Ecology* **1983**, *64*, 971–978.
22. Paini, D. Impact of the introduced honey bee (Apis mellifera)(Hymenoptera: Apidae) on native bees: A review. *Austral Ecol.* **2004**, *29*, 399–407.
23. Hoffmann, D.; Pettis, J.; Neumann, P. Potential host shift of the small hive beetle (Aethina tumida) to bumblebee colonies (Bombus impatiens). *Insectes Soc.* **2008**, *55*, 153–162.
24. Singh, R.; Levitt, A.; Rajotte, E.; Holmes, E.; Ostiguy, N. RNA viruses in hymenopteran pollinators: Evidence of inter-taxa virus transmission via pollen and potential impact on non-Apis hymenopteran species. *PLoS ONE* **2010**, *5*, e14357.
25. Graystock, P.; Goulson, D.; Hughes, W. The relationship between managed bees and the prevalence of parasites in bumblebees. *PeerJ* **2014**, *2*, e522.
26. Goulson, D.; Hughes, W. Mitigating the anthropogenic spread of bee parasites to protect wild pollinators. *Biol. Conserv.* **2015**, *191*, 10–19.
27. Stimec, J.; Scott-Dupree, C.; McAndrews, J. Honey bee, Apis mellifera, pollen foraging in southern Ontario. *Can. Field-Naturalist* **1997**, *111*, 454–456.

28. Morales, C.L.; Aizen, M.A. Does invasion of exotic plants promote invasion of exotic flower visitors? A case study from the temperate forests of the southern Andes. *Biol. Invasions* **2002**, *4*, 87–100.
29. Stout, J.C.; Kells, A.R.; Goulson, D. Pollination of the invasive exotic shrub Lupinus arboreus (Fabaceae) by introduced bees in Tasmania. *Biol. Conserv.* **2002**, *106*, 425–434.
30. Goulson, D.; Derwent, L. Synergistic interactions between an exotic honeybee and an exotic weed: Pollination of Lantana camara in Australia. *Weed Res.* **2004**, *44*, 195–202.
31. Simpson, S.; Gross, C.; Silberbauer, L. Broom and honeybees in Australia: An alien liaison. *Plant Biol.* **2005**, *7*, 541–548.
32. Mciver, J.; Thorp, R.; Erickson, K. Pollinators of the invasive plant, yellow starthistle (Centaurea solstitialis), in north-eastern Oregon, USA. *Weed Biol. Manag.* **2009**, *9*, 137–145.
33. Barthell, J.; Randall, J.; Thorp, R.; Wenner, A. Promotion of seed set in yellow star-thistle by honey bees: Evidence of an invasive mutualism. *Ecol. Appl.* **2001**, *11*, 1870–1883.
34. Groom, S.; Tuiwawa, M.; Stevens, M. Recent introduction of an allodapine bee into Fiji: A new model system for understanding biological invasions by pollinators. *Insect Sci.* **2015**, *22*, 532–540.
35. Miller, A.; Brosi, B.; Magnacca, K.; Daily, G.; Pejchar, L. Pollen carried by native and nonnative bees in the large-scale reforestation of pastureland in Hawai'i: Implications for pollination. *Pac. Sci.* **2015**, *69*, 67–79.
36. Morales, C.; Aizen, M. Invasive mutualisms and the structure of plant-pollinator interactions in the temperate forests of north-west Patagonia, Argentina. *J. Ecol.* **2005**, *94*, 171–180.
37. Rymer, P.; Whelan, R.; Ayre, D.; Weston, P.; Russell, K. Reproductive success and pollinator effectiveness differ in common and rare Persoonia species (Proteaceae). *Biol. Conserv.* **2005**, *123*, 521–532.
38. Traveset, A.; Richardson, D. Biological invasions as disruptors of plant reproductive mutualisms. *Trends Ecol. Evol.* **2006**, *21*, 208–216.
39. Aizen, M.A.; Morales, C.L.; Morales, J.M. Invasive mutualists erode native pollination webs. *PLoS Biol.* **2008**, *6*, 396–403.
40. Valido, A.; Rodríguez-Rodríguez, M.; Jordano, P. Interacciones entre plantas y polinizadores en el Parqur Nacional del Teide: Consecuencias ecológicas de la introducción masiva de la abeja doméstica, Apis. In *Proyectos de Investigacion en Parques Nacionales: 2007-2010*; Ramirez, L., Asensio, B., Eds.; Organismo Autónomo de Parques Nacionales: Madrid, Spain, 2011; pp. 205–231.
41. Valido, A.; Rodríguez-Rodríguez, M. Impacto de la introducción de la abeja doméstica (Apis mellifera, Apidae) en el Parque Nacional del Teide (Tenerife, Islas Canarias). *Ecosistemas* **2014**, *23*, 58–66.
42. Carman, K.; Jenkins, D. G. Comparing diversity to flower-bee interaction networks reveals unsuccessful foraging of native bees in disturbed habitats. *Biol. Conserv.* **2016**, *202*, 110–118.
43. Montero-Castaño, A.; Vilà, M. Influence of the honeybee and trait similarity on the effect of a non-native plant on pollination and network rewiring. *Funct. Ecol.* **2016,** doi:10.1111/1365-2435.12712.
44. Olesen, J.; Eskildsen, L. Invasion of pollination networks on oceanic islands: Importance of invader complexes and endemic super generalists. *Divers. Distrib.* **2002**, *8*, 181–192.
45. Gross, C.; Mackay, D. Honeybees reduce fitness in the pioneer shrub Melastoma affine (Melastomataceae). *Biol. Conserv.* **1998**, *86*, 169–178.
46. Polatto, L.; Chaud-Netto, J. Influence of Apis mellifera L.(Hymenoptera: Apidae) on the Use of the Most Abundant and Attractive Floral Resources in a Plant Community. *Neotrop. Entomol.* **2013**, *42*, 576–587.
47. Aizen, M.A.; Morales, C.L.; Vázquez, D.P.; Garibaldi, L.A.; Sáez, A.; Harder, L.D. When mutualism goes bad: Density-dependent impacts of introduced bees on plant reproduction. *New Phytol.* **2014**, *204*, 322–328.
48. Bruckman, D.; Campbell, D. Floral neighborhood influences pollinator assemblages and effective pollination in a native plant. *Oecologia* **2014**, *176*, 465–476.
49. Sáez, A.; Morales, C.; Ramos, L. Extremely frequent bee visits increase pollen deposition but reduce drupelet set in raspberry. *J. Appl. Ecol.* **2014**, *51*, 1603–1612.
50. Watts, S.; Sapir, Y.; Segal, B.; Dafni, A. The endangered Iris atropurpurea (Iridaceae) in Israel: Honey-bees, night-sheltering male bees and female solitary bees as pollinators. *Ann. Bot.* **2013**, *111*, 395–407.
51. Jha, S.; Vandermeer, J. Contrasting foraging patterns for Africanized honeybees, native bees and native wasps in a tropical agroforestry landscape. *J. Trop. Ecol.* **2009**, *25*, 13–22.
52. Roubik, D.; Wolda, H. Do competing honey bees matter? Dynamics and abundance of native bees before and after honey bee invasion. *Popul. Ecol.* **2001**, *43*, 53–62.
53. Villanueva-Gutierrez, R.; Roubik, D. Why are African honey bees and not European bees invasive? Pollen diet diversity in community experiments. *Apidologie* **2004**, *35*, 481–491.

54. Carneiro, L.; Martins, C. Africanized honey bees pollinate and preempt the pollen of Spondias mombin (Anacardiaceae) flowers. *Apidologie* **2012**, *43*, 474–486.
55. Roubik, D.; Villanueva-Gutierrez, R. Invasive Africanized honey bee impact on native solitary bees: A pollen resource and trap nest analysis. *Biol. J. Linn. Soc.* **2009**, *98*, 152–160.
56. Santos, G.; Aguiar, C.; Genini, J.; Martins, C. Invasive Africanized honeybees change the structure of native pollination networks in Brazil. *Biol. Invasions* **2012**, *14*, 2369–2378.
57. Villanueva-Gutiérrez, R.; Roubik, D.W.; Porter-Bolland, L. *Bee–Plant Interactions: Competition and Phenology of Flowers Visited by Bees*; Islebe, G.A., Calmé, S., León-Cortés, J.L., Schmook, B., Eds.; Springer International Publishing: Cham, Switzerland, 2015; pp. 131–152.
58. Hanley, M.; Goulson, D. Introduced weeds pollinated by introduced bees: Cause or effect? *Weed Biol. Manag.* **2003**, *3*, 204–212.
59. Goulson, D.; Hanley, M. Distribution and forage use of exotic bumblebees in South Island, New Zealand. *N. Z. J. Ecol.* **2004**, *28*, 225–232.
60. Crowther, L.; Hein, P.; Bourke, A. Habitat and forage associations of a naturally colonising insect pollinator, the tree bumblebee Bombus hypnorum. *PLoS ONE* **2014**, *9*, e107568.
61. Jones, C.; Brown, M. Parasites and genetic diversity in an invasive bumblebee. *J. Anim. Ecol.* **2014**, *83*, 1428–1440.
62. Ratti, C.; Colla, S. Discussion of the presence of an eastern bumble bee species (Bombus impatiens Cresson) in western Canada. *Pan-Pac. Entomol.* **2010**, *86*, 29–31.
63. Otterstatter, M.; Thomson, J. Does pathogen spillover from commercially reared bumble bees threaten wild pollinators? *PLoS ONE* **2008**, doi:10.1371/journal.pone.0002771.
64. Colla, S.; Otterstatter, M.; Gegear, R. Plight of the bumble bee: Pathogen spillover from commercial to wild populations. *Biol. Conserv.* **2006**, *129*, 461–467.
65. Strauss, A.; White, A.; Boots, M. Invading with biological weapons: The importance of disease-mediated invasions. *Funct. Ecol.* **2012**, *26*, 1249–1261.
66. Sachman-Ruiz, B.; Narváez-Padilla, V.; Reynaud, E. Commercial Bombus impatiens as reservoirs of emerging infectious diseases in central México. *Biol. Invasions* **2015**, *17*, 2043–2053.
67. Morales, C. Introducción de abejorros (Bombus) no nativos: Causas, consecuencias ecológicas y perspectivas. *Ecol. Austral* **2007**, *17*, 51–65.
68. Morales, C.; Arbetman, M. Rapid ecological replacement of a native bumble bee by invasive species. *Front. Ecol.* **2013**, doi:10.1890/120321.
69. Delgado, A.; Hernández, N. Informe Sobre la Situación de Bombus Ruderatus en Tenerife. Available online: http://www.interreg-bionatura.com/especies/docs/Informe%20sobre%20la%20situacion%20de%20Bombus%20ruderatus%20en%20Tenerife.pdf (accessed on 15 September 2016).
70. Schmid-Hempel, R.; Eckhardt, M. The invasion of southern South America by imported bumblebees and associated parasites. *J. Anim. Ecol.* **2014**, *83*, 823–837.
71. Ruz, L. Bee pollinators introduced to Chile: A review. In *Pollinating bees: The Conservation Link Between Agriculture and Nature*; Kevan, P.; Imperatriz-Fonseca, V., Eds.; Ministry of Environment/Brasilia, 2002; pp. 155–167.
72. Montalva, J.; Dudley, L.; Arroyo, M. Geographic distribution and associated flora of native and introduced bumble bees (Bombus spp.) in Chile. *J. Apic. Res.* **2011**, *50*, 11–21.
73. Madjidian, J.; Morales, C.; Smith, H. Displacement of a native by an alien bumblebee: Lower pollinator efficiency overcome by overwhelmingly higher visitation frequency. *Oecologia* **2008**, *156*, 835–845.
74. Matsumura, C.; Yokoyama, J.; Washitani, I. Invasion Status and Potential Ecological Impacts of an Invasive Alien Bumblebee, Bombus terrestris L. (Hymenoptera: Apidae) Naturalized in Southern Hokkaido, Japan. *Glob. Environ. Res.* **2004**, *8*, 51–66.
75. Inoue, M.N.; Yokoyama, J.; Washitani, I. Displacement of Japanese native bumblebees by the recently introduced Bombus terrestris (L.) (Hymenoptera: Apidae). *J. Insect Conserv.* **2008**, *12*, 135–146.
76. Goka, K. Introduction to the Special Feature for Ecological Risk Assessment of Introduced Bumblebees: Status of the European bumblebee, Bombus terrestris, in Japan as a. *Appl. Entomol. Zool.* **2010**, *45*, 1–6.
77. Hingston, A.; McQuillan, P. Nectar robbing in Epacris impressa (Epacridaceae) by the recently introduced bumblebee Bombus terrestris (Apidae) in Tasmania. *Vic. Nat.* **1998**, *115*, 116–119.
78. McQuillan, P.; Hingston, A. Displacement of Tasmanian native megachilid bees by the recently introduced bumblebee Bombus terrestris (Linnaeus, 1758)(Hymenoptera: Apidae). *Aust. J. Zool.* **1999**, *47*, 59–65.

79. Ings, T.; Schikora, J.; Chittka, L. Bumblebees, humble pollinators or assiduous invaders? A population comparison of foraging performance in Bombus terrestris. *Oecologia* **2005**, *144*, 508.
80. Ings, T.; Ward, N.; Chittka, L. Can commercially imported bumble bees out-compete their native conspecifics? *J. Appl. Ecol.* **2006**, *43*, 940–948.
81. Torretta, J.; Medan, D.; Abrahamovich, A. First record of the invasive bumblebee Bombus terrestris (L.)(Hymenoptera, Apidae) in Argentina. *Trans. Am. Entomol. Soc.* **2006**, *132*, 285–289.
82. noue, M.; Yokoyama, J. Competition for flower resources and nest sites between Bombus terrestris (L.) and Japanese native bumblebees. *Appl. Entomol. Zool.* **2010**, *45*, 29–35.
83. Nagamitsu, T.; Yamagishi, H.; Kenta, T.; Inari, N.; Kato, E. Competitive effects of the exotic Bombus terrestris on native bumble bees revealed by a field removal experiment. *Popul. Ecol.* **2010**, *52*, 123–136.
84. Montalva, J.; Allendes, J.; Lucia, M. The large carpenter bee Xylocopa augusti (Hymenoptera: Apidae): New record for Chile. *J. Melittology* **2013**, *12*, 1–6.
85. Nagamitsu, T.; Kenta, T.; Inari, N.; Kato, E.; Hiura, T. Abundance, body size, and morphology of bumblebees in an area where an exotic species, Bombus terrestris, has colonized in Japan. *Ecol. Res.* **2007**, *22*, 331–341.
86. Kenta, T.; Inari, N.; Nagamitsu, T.; Goka, K.; Hiura, T. Commercialized European bumblebee can cause pollination disturbance: An experiment on seven native plant species in Japan. *Biol. Conserv.* **2007**, *134*, 298–309.
87. Goka, K.; Okabe, K.; Yoneda, M.; Niwa, S. Bumblebee commercialization will cause worldwide migration of parasitic mites. *Mol. Ecol.* **2001**, *10*, 2095–2099.
88. Kanbe, Y.; Okada, I.; Yoneda, M.; Goka, K.; Tsuchida, K. Interspecific mating of the introduced bumblebee Bombus terrestris and the native Japanese bumblebee Bombus hypocrita sapporoensis results in inviable hybrids. *Naturwissenschaften* **2008**, *95*, 1003–1008.
89. Arbetman, M.; Meeus, I.; Morales, C.; Aizen, M. Alien parasite hitchhikes to Patagonia on invasive bumblebee. *Biol. Invasions* **2013**, *15*, 489–494.
90. Fürst, M. A.; McMahon, D. P.; Osborne, J. L.; Paxton, R. J.; Brown, M. J. F. Disease associations between honeybees and bumblebees as a threat to wild pollinators. *Nature* **2014**, *506*, 364–366.
91. Macfarlane, R. Fungi associated with Bombinae (Apidae: Hymenoptera) in North America. *Mycopathologia* **1976**, *59*, 41–42.
92. McFadyen, R.; Lloyd, S. Bumblebees: Implications of a new super-pollinator in mainland Australia. *Fifteenth Aust. Weeds Conf.* **2006**, *15*, 227–230.
93. Stokes, K. E.; Buckley, Y. M.; Sheppard, A. W. A modelling approach to estimate the effect of exotic pollinators on exotic weed population dynamics: Bumblebees and broom in Australia. *Divers. Distrib.* **2006**, *12*, 593–600.
94. Dafni, A.; Kevan, P.; Gross, C.; Goka, K. Bombus terrestris, pollinator, invasive and pest: An assessment of problems associated with its widespread introductions for commercial purposes. *Appl. Entomol. Zool.* **2010**, *45*, 101–113.
95. Pérez, V. Introducción de Bombus (Bombus) terrestris (Linnaeus, 1758)(Hymenoptera: Apidae) en la región de Magallanes: Potencial riesgo para las abejas nativas. *Inst. Patagon.* **2013**, *41*, 147–152.
96. Hingston, A. Does the introduced bumblebee, Bombus terrestris (Apidae), prefer flowers of introduced or native plants in Australia? *Aust. J. Zool.* **2005**, *53*, 29–34.
97. Dohzono, I.; Kunitake, Y.; Yokoyama, J.; Goka, K. Alien bumble bee affects native plant reproduction through interactions with native bumble bees. *Ecology* **2008**, *89*, 3082–3092.
98. Ishii, H.; Kadoya, T.; Kikuchi, R.; Suda, S. Habitat and flower resource partitioning by an exotic and three native bumble bees in central Hokkaido, Japan. *Biol. Conserv.* **2008**, *141*, 2597–2607.
99. Kondo, N.; Yamanaka, D.; Kanbe, Y.; Kunitake, Y. Reproductive disturbance of Japanese bumblebees by the introduced European bumblebee Bombus terrestris. *Naturwissenschaften* **2009**, *96*, 467–475.
100. Kraus, F.; Szentgyörgyi, H.; Rożej, E.; Rhode, M. Greenhouse bumblebees (Bombus terrestris) spread their genes into the wild. *Conserv. Genet.* **2011**, *12*, 187–192.
101. Ne'eman, G.; Dafni, A.; Potss, S. The effect of fire on flower visitation rate and fruit set in four core-species in east Mediterranean scrubland. *Plant Ecol.* **2000**, *146*, 97–104.

102. Hingston, B.A. The Potential Impact of the Large Earth Bumblebee "Bombus Terrestris" (Apidae) on the Australian Mainland: Lessons from Tasmania. *Vic. Nat.* **2007**, *124*, 110.
103. Dohzono, I.; Yokoyama, J. Impacts of alien bees on native plant-pollinator relationships: A review with special emphasis on plant reproduction. *Appl. Entomol. Zool.* **2010**, *45*, 37–47.
104. Crane, E. Apis species of tropical Asia as pollinators, and some rearing methods for them. *VI Int. Symp. Pollinat.* **1991**, *288*, 29–48.
105. Bhattacharya, R.; Basu, P. Pollinator Limitation and Crop Production: Experimental Observations on Few Economically Important Vegetable Crops in West Bengal, India. *Proc. Zool. Soc.* **2016**, 1–4, doi:10.1007/s12595-016-0189-4.
106. Sun, S.; Huang, S.; Guo, Y. Pollinator shift to managed honeybees enhances reproductive output in a bumblebee-pollinated plant. *Plant Syst. Evol.* **2013**, *299*, 139–150.
107. El Shafie, H.A.F.; Mogga, J.B.B.; Basedow, T. Studies on the possible competition for pollen between the honey bee, Apis mellifera sudanensis, and the imported dwarf honey bee Apis florea (Hym., Apidae) in North-Khartoum (Sudan). *J. Appl. Entomol.* **2002**, *126*, 557–562.
108. Cook, D.; Thomas, M.; Cunningham, S. Predicting the economic impact of an invasive species on an ecosystem service. *Ecol. Appl.* **2007**, *17*, 1832–1840.
109. Melin, A.; Rouget, M.; Midgley, J. Pollination ecosystem services in South African agricultural systems. *S. Afr. J. Sci.* **2014**, *110*, 1–9.
110. Ricketts, T.H.; Regetz, J.; Steffan-Dewenter, I.; Cunningham, S.A.; Kremen, C.; Bogdanski, A.; Gemmill-Herren, B.; Greenleaf, S.S.; Klein, A.M.; Mayfield, M.M.; et al. Landscape effects on crop pollination services: Are there general patterns? *Ecol. Lett.* **2008**, *11*, 499–515.
111. Freitas, B.; Imperatriz-Fonseca, V.; Medina, L. Diversity, threats and conservation of native bees in the Neotropics. *Apidologie* **2009**, *40*, 332–346.
112. Glatz, R. Curious case of the Kangaroo Island honeybee Apis mellifera Linnaeus, 1758 (Hymenoptera: Apidae) sanctuary. *Austral Entomol.* **2015**, *54*, 117–126.
113. Bjorkman, T. Role of honey bees (Hymenoptera: Apidae) in the pollination of buckwheat in eastern North America. *J. Econ. Entomol.* **1995**, *88*, 1739–1745.
114. Garibaldi, L.A.; Steffan-Dewenter, I.; Winfree, R.; Aizen, M.A.; Bommarco, R.; Cunningham, S.A.; Kremen, C.; Carvalheiro, L.G.; Harder, L.D.; Afik, O.; et al. Wild pollinators enhance fruit set of crops regardless of honey bee abundance. *Science* **2013**, *339*, 1608–1611.
115. Davis, A.R.; Solomon, K.R.; Shuel, R.W. Laboratory Studies of Honeybee Larval Growth and Development as Affected by Systemic Insecticides at Adult-Sublethal Levels. *J. Apic. Res.* **1988**, *27*, 146–161.
116. Williams. The identity of the previous visitor influences flower rejection by nectar-collecting bees. *Anim. Behav.* **1998**, *56*, 673–681.
117. Page, R.E.; Peng, C.Y.-S. Aging and development in social insects with emphasis on the honey bee, Apis mellifera L. *Exp. Gerontol.* **2001**, *36*, 695–711.
118. Grozinger, C.; Sharabash, N.; Whitfield, C.; Robinson, G. Pheromone-mediated gene expression in the honey bee brain. *Proc. Natl. Acad. Sci.* **2003**, *100*, 14519–14525.
119. Mullin, C.A.; Chen, J.; Fine, J.D.; Frazier, M.T.; Frazier, J.L. The formulation makes the honey bee poison. *Pestic. Biochem. Physiol.* **2015**, *120*, 27–35.
120. Chamberlain, S.; Schlising, R. Role of honey bees (Hymenoptera: Apidae) in the pollination biology of a California native plant, Triteleia laxa (Asparagales: Themidaceae). *Environ. Entomol.* **2008**, *37*, 808–816.
121. Junker, R.; Bleil, R.; Daehler, C. Intra-floral resource partitioning between endemic and invasive flower visitors: Consequences for pollinator effectiveness. *Ecol. Appl.* **2010**, *35*, 760–767.
122. Sanguinetti, A.; Singer, R. Invasive bees promote high reproductive success in Andean orchids. *Biol. Conserv.* **2014**, *175*, 10–20.
123. Lowenstein, D.; Matteson, K.; Xiao, I.; Silva, A. Humans, bees, and pollination services in the city: The case of Chicago, IL (USA). *Biodivers. Conserv.* **2014**, *23*, 2857–2874.
124. Giannini, T.; Garibaldi, L.; Acosta, A.; Silva, J. Native and non-native supergeneralist bee species have different effects on plant-bee networks. *PLoS ONE* **2015**, doi:10.1371/journal.pone.0137198.
125. Carvalheiro, L.G.; Veldtman, R.; Shenkute, A.G.; Tesfay, G.B.; Pirk, C.W.W.; Donaldson, J.S.; Nicolson, S.W. Natural and within-farmland biodiversity enhances crop productivity. *Ecol. Lett.* **2011**, *14*, 251–259.
126. Pinkus-Rendon, M.; Parra-Tabla, V. Floral resource use and interactions between Apis mellifera and native bees in cucurbit crops in Yucatan, Mexico. *Can. Entomol.* **2005**, *137*, 441–449.

127. Aizen, M.A.; Feinsinger, P. Habitat Fragmentation, Native Insect Pollinators, and Feral Honey Bees in Argentine "Chaco Serrano." *Ecol. Appl.* **1994**, *4*, 378–392.
128. Gurr, L. The role of bumblebees as pollinators of red clover and lucerne in New Zealand: A review and prospect. *Proc. New Zeal. Grassl. Assoc.* **1974**, *36*, 111–122.
129. McCarthy, B. *Habitat Use by Bumble Bees (Hymenoptera: Apidae: Bombus spp.) in New Zealand*; Lincoln University: Oakland, CA, USA, 2006.
130. Ellis, J.; Knight, M.; Goulson, D. Delineating species for conservation using mitochondrial sequence data: the taxonomic status of two problematic Bombus species (Hymenoptera: Apidae). *J. Insect Conserv.* **2005**, *9*, 75–83.
131. Benazzo, A.; Ghirotto, S.; Vilaca, S.; Hoban, S. Using ABC and microsatellite data to detect multiple introductions of invasive species from a single source. *Heredity (Edinb)* **2015**, *115*, 262–272.
132. Lye, G.; Kaden, J.; Park, K.; Goulson, D. Forage use and niche partitioning by non-native bumblebees in New Zealand: Implications for the conservation of their populations of origin. *J. insect Conserv.* **2010**, *14*, 607–615.
133. Lye, G.; Lepais, O.; Goulson, D. Reconstructing demographic events from population genetic data: The introduction of bumblebees to New Zealand. *Mol. Ecol.* **2011**, *20*, 2888–2900.
134. Goulson, D.; Williams, P. Bombus hypnorum (L.)(Hymenoptera: Apidae), a new British bumblebee? *Br. J. Entomol. Nat. Hist.* **2001**, *14*, 129–131.
135. Prŷs-Jones, O. The Tree Bumble Bee (Bombus hypnorum) as a House Sparrow equivalent? Comments on colonizing success in Britain in the context of declining native species. *Bee World* **2014**, *91*, 98–101.
136. Artz, D.R.; Nault, B.A. Performance of Apis mellifera, Bombus impatiens, and Peponapis pruinosa (Hymenoptera: Apidae) as pollinators of pumpkin. *J. Econ. Entomol.* **2011**, *104*, 1153–1161.
137. Vaudo, A.D.; Patch, H.M.; Mortensen, D.A.; Tooker, J.F.; Grozinger, C.M. Macronutrient ratios in pollen shape bumble bee (Bombus impatiens) foraging strategies and floral preferences. *Proc. Natl. Acad. Sci. USA* **2016**, *113*, E4035-42.
138. MacIvor, J. Wild Bees in Cultivated City Gardens. In *Sowing Seeds in the City*; Brown, S., McIvor, K., Snyder, E., Eds.; Springer Science: New York, NY, USA, 2016; pp. 207–227.
139. Arretz, P.; Macfarlane, R. The introduction of Bombus ruderatus to Chile for red clover pollination. *Bee World* **1986**, *67*, 15–22.
140. Pomeroy, N. Use of natural sites and field hives by a long-tongued bumble bee Bombus ruderatus. *N. Z. J. Agric. Res.* **1981**, *24*, 409–414.
141. Bartlett, M.; Hale, R.; Hale, M. Habitat quality limits gene flow between populations of Bombus ruderatus in the South Island, New Zealand. *Conserv. Genet.* **2016**, *17*, 703–713.
142. Acosta, A.; Giannini, T.; Imperatriz-Fonseca, V. Worldwide alien invasion: A methodological approach to forecast the potential spread of a highly invasive pollinator. *PLoS ONE* **2016**, *11*, e0148295.
143. Geslin, B.; Morales, C. New records reveal rapid geographic expansion of Bombus terrestris Linnaeus, 1758 (Hymenoptera: Apidae), an invasive species in Argentina. *Check List* **2015**, *11*, 1620.
144. Ayasse, M.; Jarau, S. Chemical ecology of bumble bees. *Annu. Rev. Entomol.* **2014**, *59*, 299–319.
145. Vaudo, A.D.; Stabler, D.; Patch, H.M.; Tooker, J.F.; Grozinger, C.M.; Wright, G.A. Bumble bees regulate their intake of the essential protein and lipid pollen macronutrients. *J. Exp. Biol.* **2016**, doi:10.1242/jeb.140772.
146. Banaszak-Cibicka, W.; Żmihorski, M. Wild bees along an urban gradient: Winners and losers. *J. Insect Conserv.* **2012**, *16*, 33.

© 2016 by the authors; licensee MDPI, Basel, Switzerland. This article is an open access article distributed under the terms and conditions of the Creative Commons by Attribution (CC-BY) license (http://creativecommons.org/licenses/by/4.0/).
